# Unveiling the Structure of Anhydrous Sodium Valproate with 3D Electron Diffraction and a Facile Sample Preparation Workflow

**DOI:** 10.1021/acscentsci.5c00412

**Published:** 2025-05-21

**Authors:** Jiaoyan Xu, Vivek Srinivas, Rohit Kumar, Laura Pacoste, Yiwang Guo, Taimin Yang, Changquan Calvin Sun, Martin Högbom, Xiaodong Zou, Hongyi Xu

**Affiliations:** † Department of Chemistry, 7675 Stockholm University, SE-106 91 Stockholm, Sweden; ‡ Department of Biochemistry and Biophysics, Stockholm University, SE-106 91 Stockholm, Sweden; § 5635 University of Minnesota, WDH 9-177, 308 Harvard St. S.E., Minneapolis, Minnesota 55455, United States; ∥ Research School of Chemistry, Australian National University, Acton ACT 2601, Australia

## Abstract

Understanding the structure of an active pharmaceutical ingredient is essential for gaining insights into its physicochemical properties. Sodium valproate, one of the most effective antiepileptic drugs, was first approved for medical use in 1967. However, the structure of its anhydrous form has remained unresolved. This is because it was difficult to grow crystals of sufficient size for single-crystal X-ray diffraction (SCXRD). Although 3D electron diffraction (3D ED) can be used for studying crystals that are too small for SCXRD, the crystals of anhydrous sodium valproate are extremely sensitive to both humidity and electron beams. They degrade quickly both in air and under an electron beam at room temperature. In this study, we developed a glovebox-assisted cryo-transfer workflow for the preparation of EM grids in a protected atmosphere to overcome the current challenges for studying air- and beam-sensitive samples using 3D ED. Using this technique, we successfully determined the structure of anhydrous sodium valproate, revealing the formation of Na-valproate polyhedral chains. Our results provide a robust framework for the 3D ED analysis of air-sensitive crystals, greatly enhancing its utility across various scientific disciplines.

## Graphical abstract

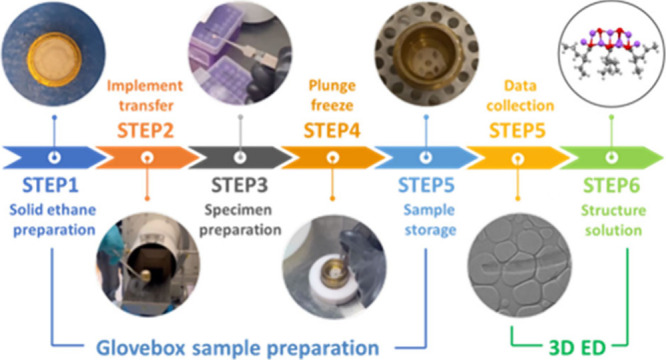

## 1 Introduction

Valproates, a family of widely effective antiepileptic drugs (AED), have demonstrated remarkable efficacy in treating various seizures and epileptic syndromes.^1,2^ Multiple clinical studies have indicated that valproates exhibit the broadest spectrum of anticonvulsant activity among all currently available AEDs across all age groups.^3^ The term “valproates” refers to a group of related compounds that release valproate ions in the plasma, with the most common compounds being valproic acid and sodium valproate. Valproic acid (VPA), the original compound, was synthesized in 1882 ^4^ and its antiepileptic properties were discovered in 1962.^5^ Since its introduction to the market in the late 20th century,^6^ VPA has progressively became a primary treatment for conditions ranging from epilepsy to cancer.^7–10^ Despite its high effectiveness, the nonionized molecular structure of VPA results in poor water solubility,^11^ which complicates drug formulation^12^ and potentially leads to gastrointestinal irritation upon administration.^13^ To overcome these issues, sodium valproate was developed.^14^ This derivative maintains the therapeutic effects^15^ of valproic acid while providing improved bioavailability^16^ and reduced risk of gastrointestinal irritation because of its higher solubility.^17^

To better understand its physicochemical properties, it is essential to determine the crystal structure of sodium valproate. However, sodium valproate exhibits very high hygroscopicity,^17^ which presents a significant challenge for its structure determination. As shown in Figure 1, sodium valproate deliquesced when exposed to a relative humidity (RH) of above 40%, making sample preparation under ambient conditions difficult. According to a previous report,^18^ seven stable polymorphs of sodium valproate have been identified, but only the crystal structure of a monohydrate form was determined using single-crystal X-ray diffraction (SCXRD) under cryogenic conditions.^19^ Attempts to solve the structures of the other polymorphs and crystal forms using X-ray diffraction have been unsuccessful, as even the cell parameters could not be determined with the application of high-intensity synchrotron X-ray radiation, which typically requires well-ordered crystals larger than 5 × 5 × 5 μm^3^.^20^ This failure is primarily attributed to the poor crystallinity of the compound, limited crystal size, and high hygroscopicity of the compound, which require a humidity-free environment to prevent deliquescence during analysis. Furthermore, the PXRD pattern collected under dry conditions (Figure S1, experimental details provided in the Methods) shows peak broadening and overlapping, making *ab initio* structure solution of anhydrous sodium valproate by PXRD challenging.

**Figure 1:**
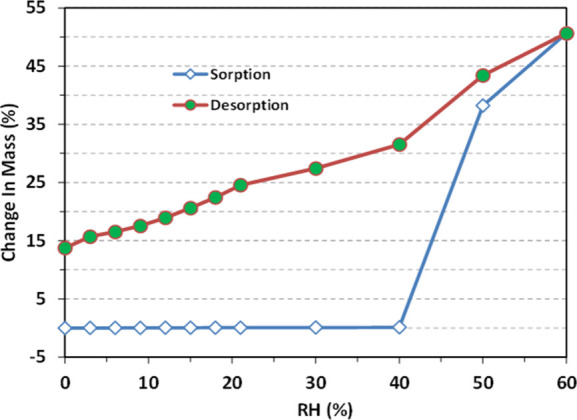
Moisture sorption isotherm of sodium valproate at 298 K. A sharp increase in mass above 40% RH indicates deliquescence of sodium valproate. The details of the hygroscopicity measurements are provided in the Methods.

Owing to the strong interaction between electrons and matter, 3D ED can determine crystal structures from samples that are too small for SCXRD or too complex for powder X-ray diffraction.^21,22^ However, this strong interaction also renders the sample vulnerable to electron beam damage.^23,24^ To mitigate this issue during data collection, cryogenic protection is essential to preserve the sample against the electron beam.^23^

To overcome the dual sensitivity of anhydrous sodium valproate to humidity and electron beams, it is essential to develop a robust workflow that preserves the crystals during specimen preparation and 3D ED data collection.^25,26^ In this study, a nitrogen-regulated glovebox (Figure 2) equipped with a cooling chamber was designed to enable plunge freezing^27^ in a controlled atmosphere (Video S1). This specialized setup enabled the anhydrous sodium valproate crystals to be preserved and transferred without air exposure, thus enabling their structure determination. Our method offers significant advantages in preserving sample integrity and enhancing experimental efficiency. By enabling plunge freezing, the prepared sample can be rapidly frozen into its *in situ* state, allowing for electron crystallography experiments without compromising sample stability. Additionally, unlike approaches that require transferring the entire sample preparation device and holder into a glovebox, our method ensures broader compatibility with different setups while maintaining a controlled environment. Furthermore, our approach enhances throughput by enabling the preparation of multiple samples in a single procedure. Beyond its application to sodium valproate, this workflow is broadly applicable to other air-sensitive materials, including battery components, semiconductors, and biological samples. As many of these materials are also highly susceptible to electron beam damage, cryogenic protection is essential to minimize beam-induced degradation during the 3D ED data collection.

**Figure 2:**
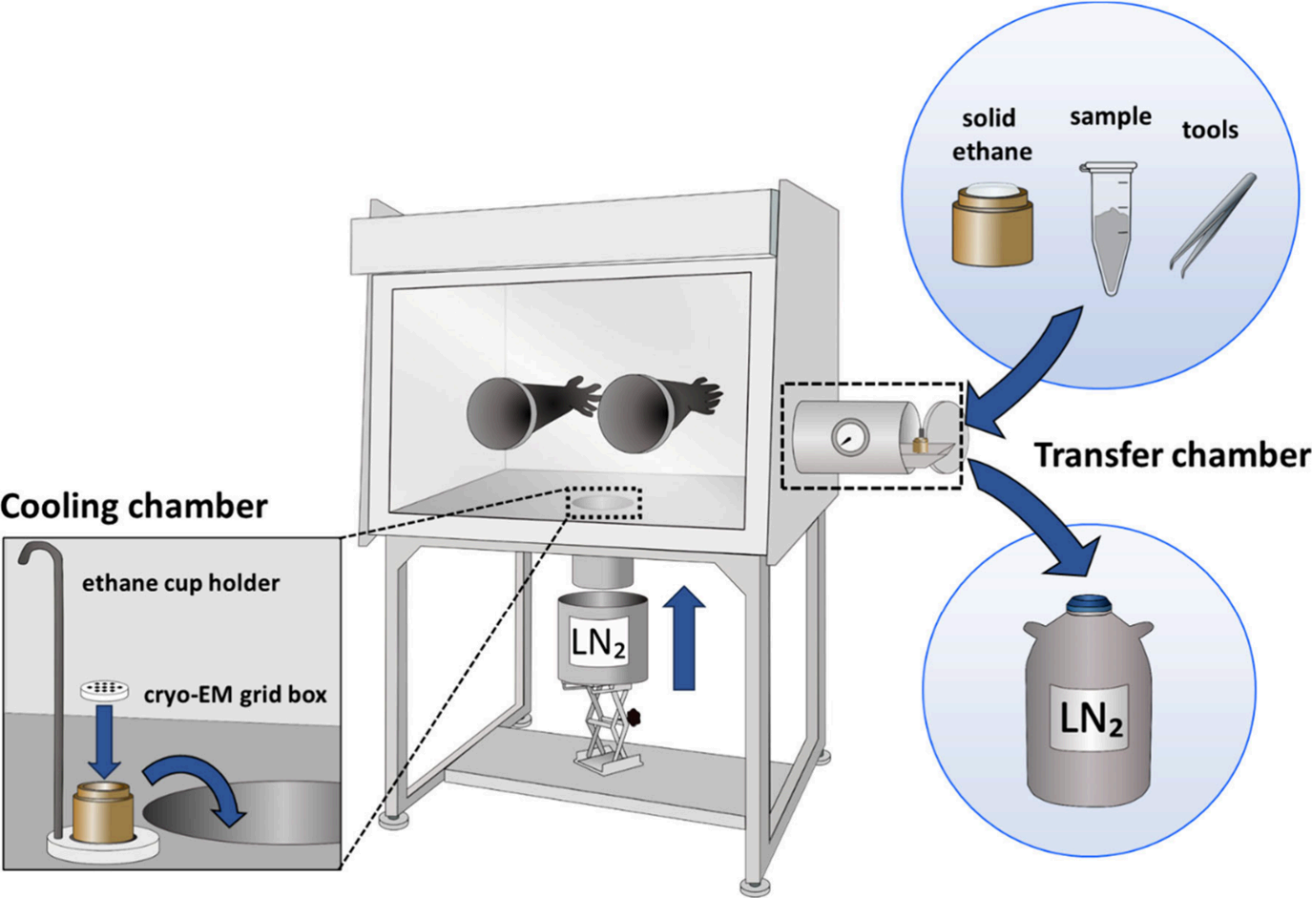
Overview of a nitrogen-regulated glovebox designed for plunge freezing. The schematic design and a photograph of the glovebox setup are shown in Figures S2 and S3.

## Result and Discussion

### Plunge Freezing of Sodium Valproate Crystals in a Glovebox

Lacey carbon film supported copper TEM grids were glow-discharged for 60 s at 20 mA (PELCO easiGLOW) to make the carbon supporting film hydrophilic. The pretreated grids, along with sodium valproate and essential tools, were then transferred into the nitrogen-regulated glovebox through the transfer chamber. The cooling chamber of the glovebox was precooled to liquid nitrogen (LN_2_) temperature using an LN_2_-filled bucket (Figure 2).

To preserve the cryogen during transfer into the glovebox, it is crucial to first freeze ethane into a solid state before loading it into the transfer chamber. Ethane gas is first condensed into liquid in a copper cryogen cup and cooled by LN_2_. To solidify the liquid ethane, we carefully placed the ethane-filled cup in a bath of LN_2_. It is critical to ensure that the cup is surrounded by LN_2_, but not fully submerged in the LN_2_ bath. This gradual cooling from the sides minimizes the splashing during the freezing process. Once the ethane solidified, the cup was fully submerged in the LN_2_ bath. Transferring ethane in its solid state significantly reduces mass loss under the low-pressure conditions of the transfer chamber (Figure 2) compared to transferring it as a liquid. Additionally, placing tissue beneath the ethane-filled cup reduces heat transfer, thereby minimizing the melting and evaporation of ethane. After being transferred into the glovebox, solid ethane gradually melts back into the liquid phase, ready for plunge freezing.

Prior to sample preparation, a cryo-EM grid box is placed into an ethane-filled cup to facilitate downstream cryo-transfer of TEM grids. The ethane cup was then positioned on a 3D-printed cup holder and stored in the cooling chamber, allowing the ethane cup to be conveniently lowered into and retrieved from the cooling chamber (Figure 2) when not in use. The cooling chamber significantly reduces ethane evaporation during other necessary specimen preparation procedures before plunge freezing. Additionally, the ethane cup holder isolates the cryogen cup from the metal bench of the glovebox, further minimizing cryogen loss by reducing the heat transfer.

Under the protection of the humidity-free nitrogen atmosphere in the glovebox, the anhydrous sodium valproate crystals were crushed between two glass slides into submicrometer crystal fragments. These fragments were then transferred onto a glow-discharged TEM grid by gently dipping the grid into the fragments. Next, the ethane-filled cup was lifted from the cooling chamber, and the grid was manually plunged into liquid ethane. The plunge-frozen grid was then placed into a slot of the cryo-EM grid box kept at the bottom of the ethane-filled cup. Once several grids were prepared and stored, the entire ethane-filled cup, containing the cryosample box with the plunge-frozen grids, was transferred out of the glovebox. Finally, the entire setup was stored in liquid nitrogen for storage. For 3D ED data collection, prepared grids were cryotransferred onto a side-entry cryo-transfer holder (Gatan Elsa) and inserted into a transmission electron microscope (ThermoFisher Scientific Themis Z). The workflow is illustrated in Scheme 1.

**Scheme 1:**
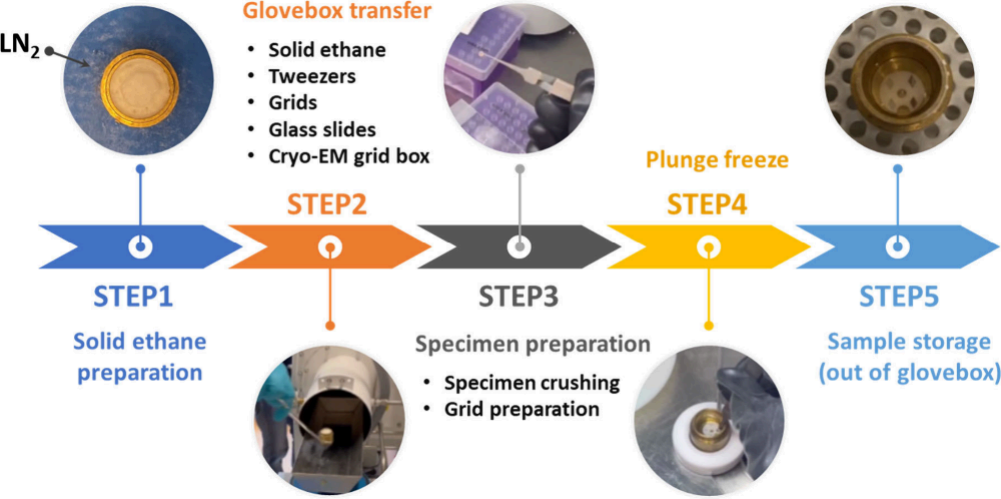
Overall Workflow of the Glove-Boxed Assisted Cryo-transfer Sample Preparation for 3D ED^a^

a A video showing the sample preparation workflow is available in the Supporting Information.

### Structure Determination of Sodium Valproate

Owing to its high hygroscopicity, anhydrous sodium valproate crystal deliquesces and loses its crystallinity rapidly during sample preparation under an ambient environment. To demonstrate this effect, the sample was crushed and plunge-frozen on a conventional lab bench. As shown in Figure 3a, the samples prepared under ambient environment exhibited thawed morphology. Additionally, no reflections were observed in the electron diffraction pattern (Figure 3a, inset), indicating a loss of crystallinity. On the other hand, by using the cryo-transfer workflow introduced earlier, the crystallinity of anhydrous sodium valproate was successfully preserved. As shown in Figure 3b, a ribbon-like morphology of crystal was observed. High quality electron diffraction patterns could be collected from these crystals, confirming the effectiveness of our sample preparation workflow.

**Figure 3:**
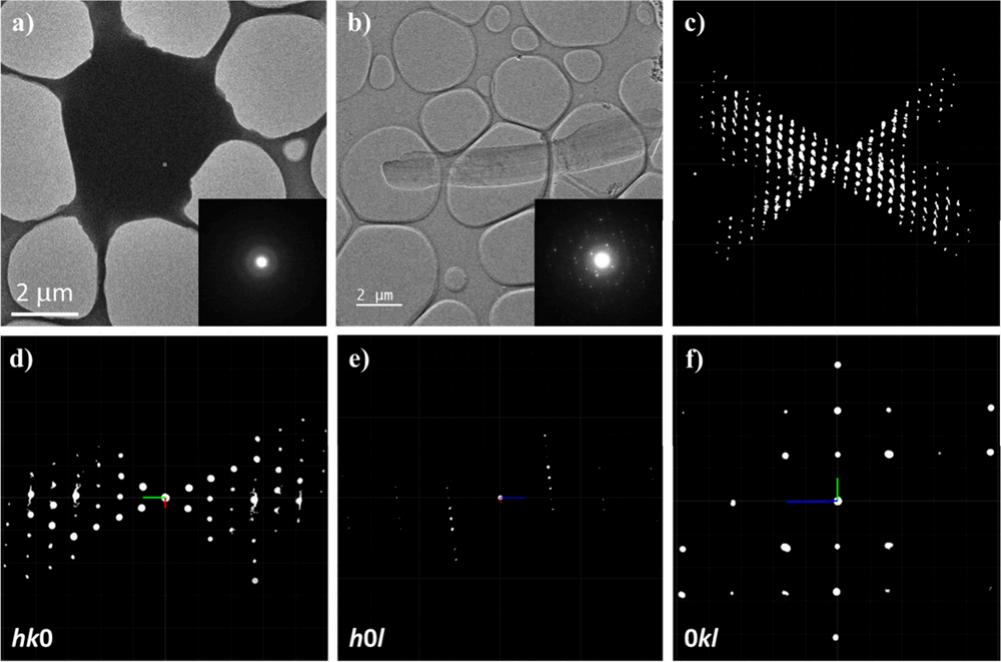
(a, b) TEM bright-field (BF) images and corresponding selected area electron diffraction (SAED) patterns (insets) of sodium valproate prepared under ambient and cryo-glovebox conditions, respectively. (c) Reconstructed 3D reciprocal lattice of sodium valproate. (d–f) 2D slices extracted from the 3D reciprocal lattice, corresponding to the (*hk*0), (*h*0*l*), and (0*kl*) planes, respectively. The unit cell parameters in reciprocal space a*, b*, and c* are highlighted in red, green, and blue, respectively.

Several 3D ED data sets were collected from anhydrous sodium valproate crystals (Figure 3c). Based on the reconstructed reciprocal lattice, the crystal system was determined as monoclinic with unit cell parameters: *a* = 31.06(6) Å, *b* = 14.36(3) Å, *c* = 6.24(12) Å, and β = 95.28(3)°. As shown in Figure 3d–f, the reflection conditions of anhydrous sodium valproate crystals were *hkl*: *h* + *k* = 2*n*, *h*0*l*: *h*, *l* = 2*n*, and 0*k*0: *k* = 2*n*, indicating that its space group is *Cc* (no. 9) or *C*2*/c* (no. 15). Structure solutions in the *C*2/*c* space group failed to yield a chemically reasonable model and resulted in disorder and steric clashes. In contrast, solution and refinement in the *Cc* space group produced a chemically reasonable structure model. Accordingly, the initial structure was solved in *Cc* from the integrated intensities using SHELXT^28^ and subsequently refined by SHELXL.^29,30^

The crystal structure reveals that anhydrous sodium valproate crystallizes in the noncentrosymmetric monoclinic space group *Cc*, with an asymmetric unit (Figure 4a) formulated as Na_3_(valp)_3_ [valp = valproate]. The sodium cations coordinate with negatively charged carboxylate groups (−COO^–^) through oxygen atoms, forming a one-dimensional coordinated chain along the *c*-axis (Figure 4b), which is stabilized by noncovalent intermolecular interactions. In the structure, one type of sodium cation (Na1) is six-coordinate, adopting a slightly distorted trigonal prism geometry. As shown in Figure 4c, the coordination sphere of Na1 is completed by six oxygen atoms, four of which come from four different valproate ions, while the remaining two oxygen atoms come from the same valproate ion. The other types of sodium cations (Na2, Na3) are five-coordinated, and each exhibits a distorted square pyramidal geometry, as shown in Figure 4d. These five coordination sites are oxygen atoms from four different valproate ions, two of which come from the same valproate ion. All of these coordinations exhibit distortions from ideal geometries, resulting in a more flexible environment around the sodium ions (Figure 4e). This flexibility facilitates the potential for water molecules to easily enter the structure and coordinate with the sodium ions, consequently influencing its structural and chemical properties.

**Figure 4:**
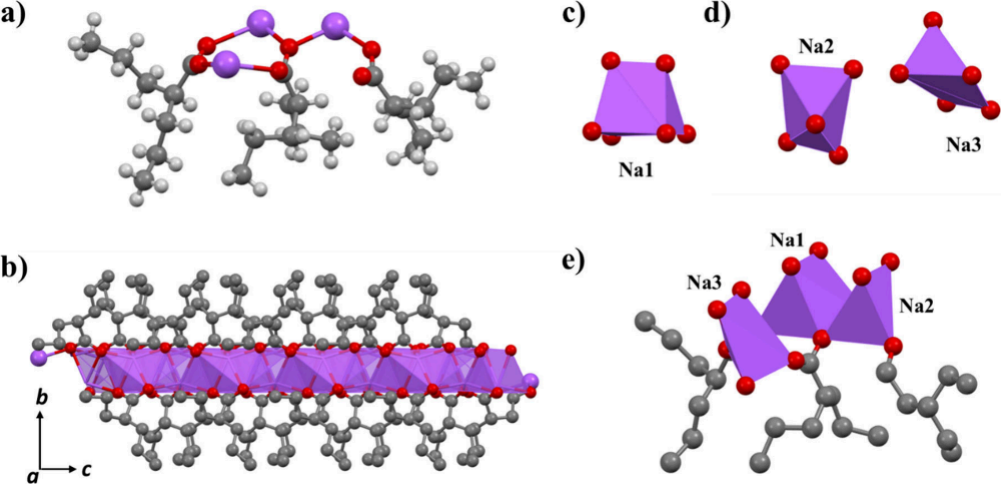
Structure model of anhydrous sodium valproate. (a) Asymmetric unit of anhydrous sodium valproate. Color code: purple, sodium; red, oxygen; gray, carbon; light gray, hydrogen. (b) Fragment of the crystal packing of anhydrous sodium valproate viewed along *a* axis with sodium cations presented in polyhedral form. (c) Coordination environment of six-coordinate sodium cation in anhydrous sodium valproate. (d) Coordination environments of five-coordinate sodium cations in anhydrous sodium valproate. (e) Coordination mode of sodium cations with valproate ions in anhydrous sodium valproate.

As shown in Figure 5, the nonpolar alkyl chains of valproate ions interact via London dispersion forces, contributing to the overall molecular packing and intrinsic stability that sustain the crystal lattice. However, due to the weak nature of London dispersion forces, water molecules may diffuse into the interalkyl spaces when sodium valproate is exposed to air. Meanwhile, the hydrophilic sodium ion provides potential binding sites for water molecules. This process weakens the ionic interactions and van der Waals force that maintain the structure integrity of anhydrous sodium valproate. Meanwhile, the highly distorted coordination of sodium ions makes the ionic interactions more susceptible to water binding, ultimately leading to the deliquescence of sodium valproate.

**Figure 5:**
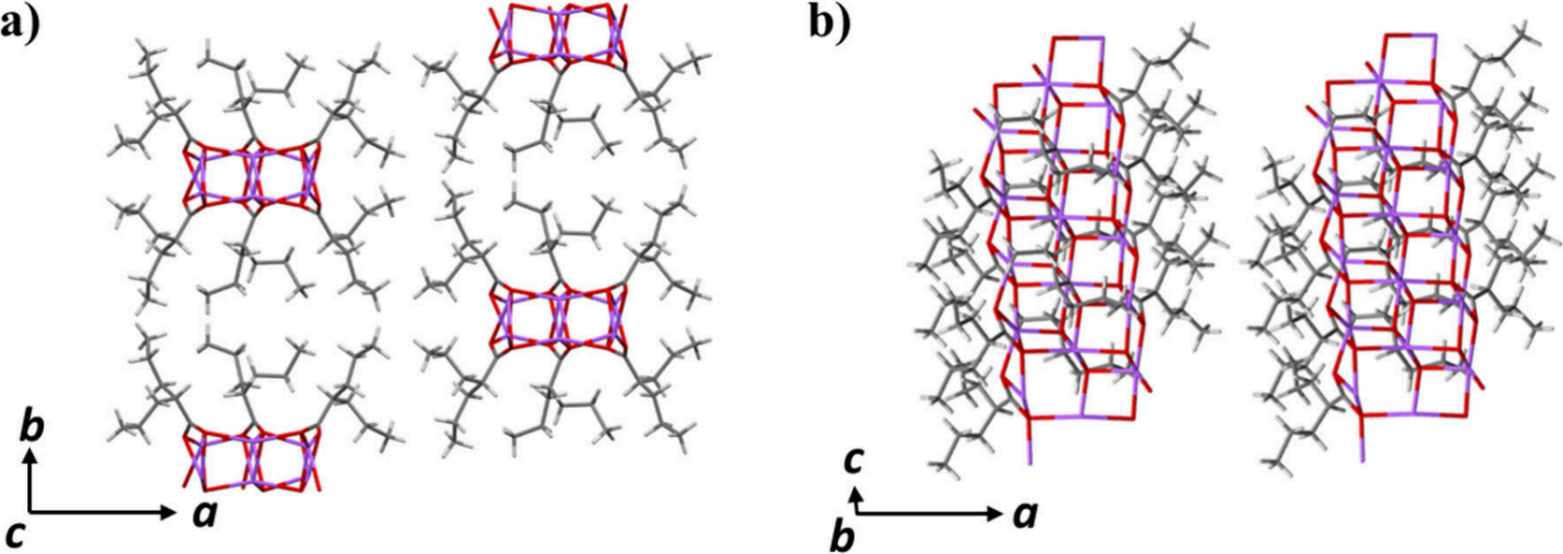
Crystal packing of anhydrous sodium valproate along the (a) *c*-axis and (b) *b*-axis. Color code: purple, sodium; red, oxygen; gray, carbon; light gray, hydrogen.

### Monohydrate and Anhydrous Sodium Valproate

The determined structure of anhydrous sodium valproate provides detailed information about its coordination environment and inter/intramolecular interactions. Comparing its structure with the monohydrate form is crucial to understanding potential differences in physicochemical properties, which may influence the pharmaceutical performance. As shown in Figure 6a, the monohydrate form crystallizes in the triclinic space group *P*-1, with a water molecule coordinating to the sodium ion. Similar to the anhydrous form, the monohydrate form is stabilized by van der Waals interactions between the alkyl chains of the valproate molecules. The sodium–oxygen coordination clusters of the monohydrate form and anhydrous form are shown in Figures 6b and 6c, respectively. The anhydrous form exhibits a more compact and regular atomic arrangement compared to that of the monohydrate form. The calculated density of the anhydrous form is 1.195 g/cm^3^, whereas the monohydrate form has a lower density of 1.099 g/cm^3^.

**Figure 6:**
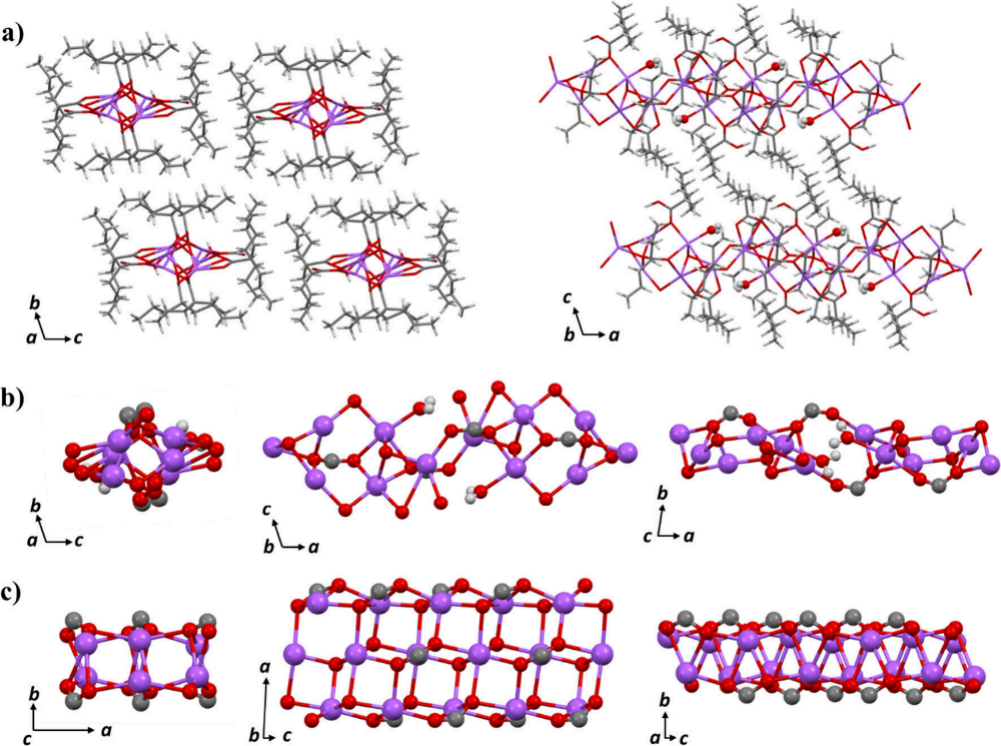
(a) Crystal packing of monohydrate sodium valproate along the *a*-axis and the *b*-axis. Color code: purple, sodium; red, oxygen; gray, carbon; light gray, hydrogen. (b) 3D structure of the sodium–oxygen clusters in monohydrate sodium valproate along the *a*-axis, *b*-axis, and *c*-axis. (c) 3D structure of the sodium–oxygen clusters in anhydrous sodium valproate along the *c*-axis, *b*-axis, and *a*-axis.

## Conclusions

The cryo-transfer sample preparation workflow developed in this study effectively preserved the integrity of anhydrous sodium valproate crystals for 3D ED analysis, allowing for the successful determination of its structure. This, in turn, provides a comprehensive understanding of the hygroscopicity of sodium valproate, which will facilitate its therapeutic applications and pharmaceutical research. By utilizing the humidity-free atmosphere of the glovebox, the samples were protected from ambient moisture, effectively prevented moisture uptake and subsequent deliquescence during specimen preparation. Furthermore, plunge freezing enabled sample analysis under cryogenic conditions, reducing beam damage during data collection. This workflow not only facilitated the successful structure determination of anhydrous sodium valproate but also offers the potential for the discovery and structure determination of other crystal forms. When combined with controlled crystallization conditions, such as variations in humidity and temperature, this approach could enable the isolation and structure characterization of additional sodium valproate polymorphs and crystal forms, including metastable forms that are otherwise challenging to capture. Such advancements will provide deeper insights into the polymorphism of sodium valproate and its impact on material properties, which are valuable for optimizing formulation and manufacturing processes for sodium-valproate-based drug products. Furthermore, this method could be used for sample preparation of other sensitive materials. It is a valuable tool for detailed structure studies across a wide range of materials including batteries, semiconductors, and biological samples.

## Methods

### Material

Anhydrous sodium valproate (C_8_H_15_NaO_2_, Figure 7) was purchased from Sigma-Aldrich (St. Louis, MO, USA), and stored in a glovebox to protect it from moisture.

**Figure 7:**
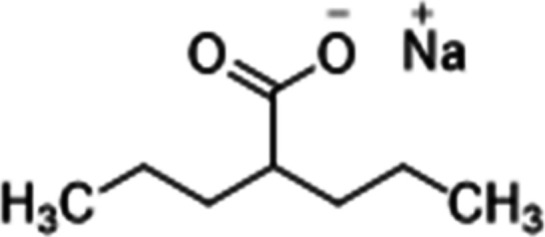
Molecular structure of sodium valproate.

### Power X-ray Diffraction

Powder X-ray diffraction (PXRD) data were collected on a PANalytical X’Pert PRO diffractometer equipped with a Cu Kα radiation source (λ = 1.5406 Å) operating at 45 kV and 40 mA. The measurements were performed in reflection mode using a PW3064/60 spinner stage. Data were recorded over a 2θ range of 5.00–35.00° with a step size of 0.0167° and a continuous scan mode. A fixed divergence slit of 0.10 mm was used. To prevent deliquescence of the sample, the relative humidity was strictly controlled to remain below 40% throughout the PXRD measurement.

### 3D Electron Diffraction

3D ED data were collected on a Themis Z microscope (300 kV) equipped with a Gatan OneView IS detector. A Gatan Elsa 698 cryo-transfer tomography holder was used for data collection. 3D ED data were collected in TEM mode using a selected-area aperture. Individual crystals were identified manually and rotated continuously at a rate of 2.7° s^–1^ via the control of *Instamatic* software.^31^ The electron flux used for data collection was 0.007 e^–1^ Å^–2^ s^–1^. A total of 32 data sets were acquired from different crystals with an exposure time of 0.1 s per frame. The 3D reciprocal lattice was reconstructed from the recorded 2D diffraction patterns using RED*p*.^32^ The space group was determined based on reflection conditions.

XDS (X-ray Detector Software)^33^ was used for unit cell determination (summarized in Table S1), as well as indexing reflections and integrating their intensities. A total of 10 data sets were scaled using XSCALE and merged based on their pairwise correlation coefficients to improve the completeness and *I*/σ­(*I*). It is worth noting that diffraction patterns collected along major zone axes were excluded during data processing to eliminate reflections severely affected by dynamical scattering. To improve data redundancy, 3D ED data sets were merged from multiple crystals. By combining data from crystals of varying thicknesses and orientations, the accuracy of the measured reflection intensities was further enhanced.^34^ The initial model of sodium valproate was solved from the merged data set by dual-space methods using SHELXT.^28^ However, due to the relatively high mosaicity of the crystals and presence of dynamical scattering in 3D ED data, the refinement did not yield chemically meaningful anisotropic displacement parameters. Therefore, the structure was refined using SHELXL^30^ and ShelXle^29^ with isotropic atomic displacement parameters. The structure was deposited in the Cambridge Crystallographic Data Centre and can be accessed by CCDC 2356987. Structure solution and refinement statistics are listed in Table S2.

### Hygroscopicity Measurement

The moisture sorption isotherm was collected using an automated vapor sorption analyzer (Intrinsic DVS, Surface Measurement Systems, Ltd., Allentown, PA, USA) at 25 °C with a nitrogen flow rate of 50 mL/min. Sample weight was monitored by a micro balance. The sample was first purged with dry nitrogen until a constant weight was obtained. During sorption, the RH was varied from 0% to 21% in 3% increments and then from 30% to 60% in 10% increments. During desorption, RH was decreased from 60% to 0% in the reverse order of sorption. Samples were equilibrated at each step with the equilibration criteria of either d*m*/d*t* = 0.003% or a maximum equilibration time of 6 h was reached before changing to the next target RH.

## Supplementary Material







## Data Availability

CCDC 2356987 contains the crystallographic data for this paper. These data can be obtained free of charge via www.ccdc.cam.ac.uk/data_request/cif, or by emailing data_request@ccdc.cam.ac.uk, or by contacting The Cambridge Crystallographic Data Centre, 12 Union Road, Cambridge CB2 1EZ, UK; fax: + 44 1223 336033.

## Notes

Figure S1, experimental PXRD patterns of anhydrous sodium valproate; Figure S2, schematic design of the nitrogen-regulated glovebox equipped with a cooling stage; Figure S3, a photograph of the nitrogen-regulated glovebox with cooling stage; Table S1, summary of the unit cell parameters of anhydrous sodium valproate crystals determined by 3D ED; and Table S2, structure solution and refinement statistics of anhydrous sodium valproate (PDF)
Video S1, workflow for cryo-sample preparation using plunge freezing in a glovebox (MP4)
Transparent Peer Review report available (PDF)
