# Supplementary material for: Unveiling the Structure of Anhydrous Sodium Valproate with 3D Electron Diffraction and a Facile Sample Preparation Workflow
Source: ACS Cent Sci. 2025 May 21;11(6):960–6. doi: 10.1021/acscentsci.5c00412 (PMC12203429; doi:10.1021/acscentsci.5c00412)
Supplement: Supplementary file 1 [file oc5c00412_si_001.pdf]

Supporting information for

Unveiling the Structure of Anhydrous Sodium Valproate with 3D Electron Diffraction and a Facile Sample Preparation Workflow

Jiaoyan Xu,[a] Vivek Srinivas,[b] Rohit Kumar,[b] Laura Pacoste,[a] Yiwang Guo,[c] Taimin Yang,[a] Changquan Calvin Sun,[c] Martin Högbom,[a] Xiaodong Zou[a]*and Hongyi Xu[a,d]*

a Department of Chemistry, Stockholm University, SE-106 91 Stockholm, Sweden

b Department of Biochemistry and Biophysics, Stockholm University, SE-106 91 Stockholm, Sweden

c University of Minnesota, WDH 9-177, 308 Harvard St. S.E. Minneapolis, MN 55455, U.S.A.

d Research School of Chemistry, Australian National University, Acton, ACT 2601, Australia.

E-mail: hongyi.xu@su.se, xiaodong.zou@su.se.

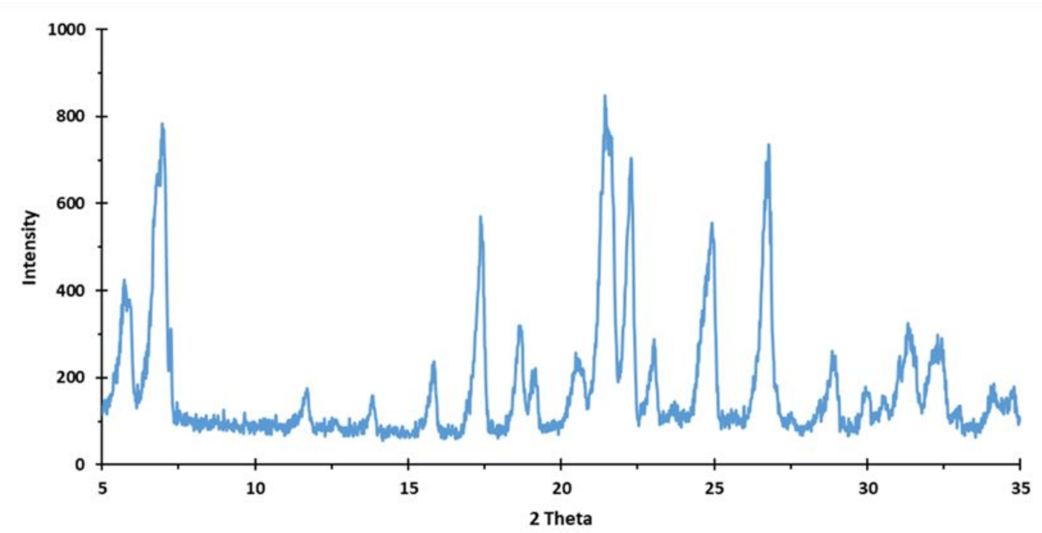

Figure S1. Experimental PXRD pattern of anhydrous sodium valproate.

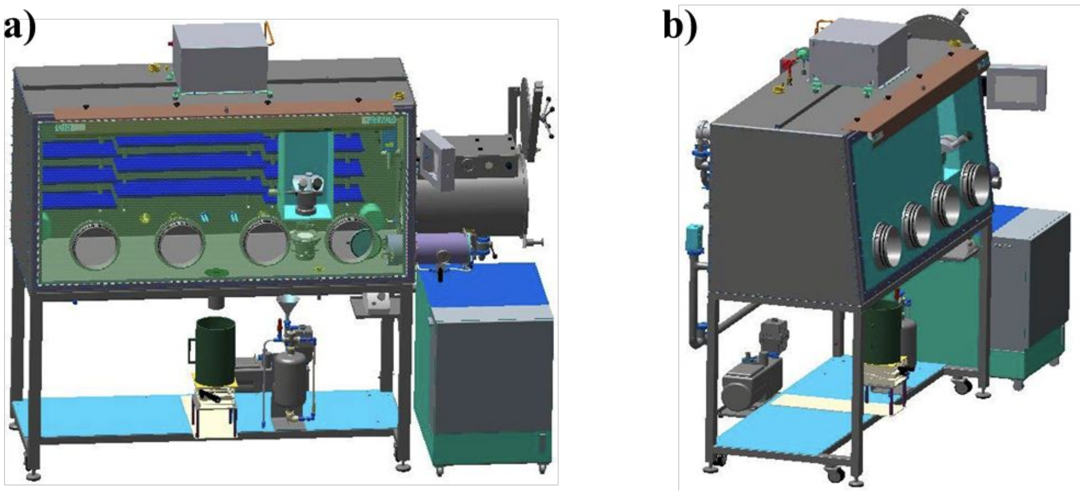

Figure S2. Schematic design of the nitrogen-regulated glovebox equipped with a cooling stage.

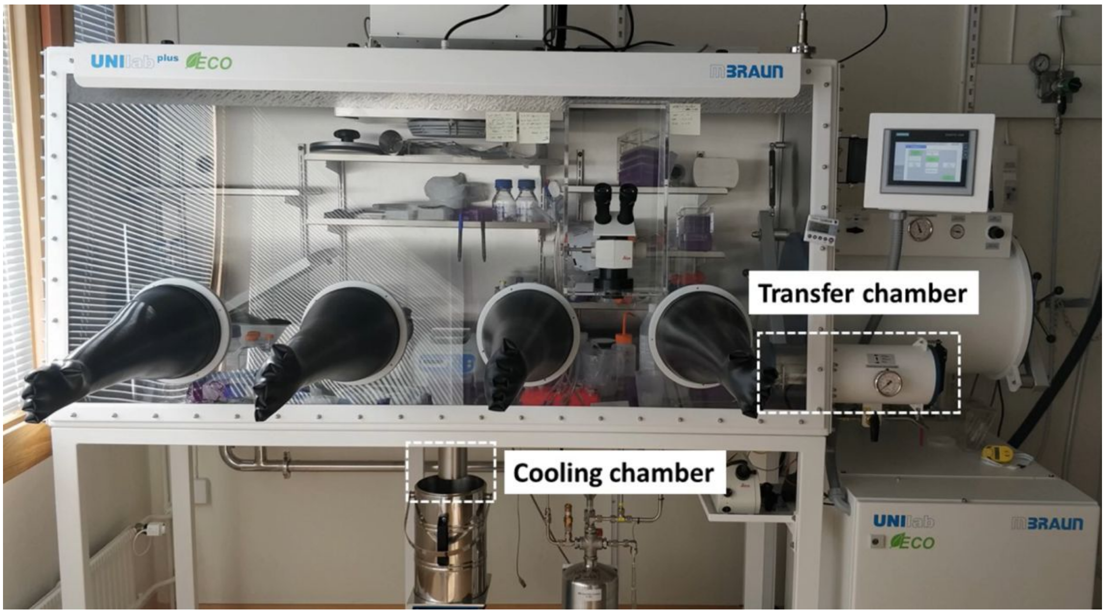

Figure S3. A photograph of the nitrogen-regulated glove box with cooling stage.

Table S1. Summary of the unit cell parameters of anhydrous sodium valproate crystals determined by 3D ED.

Dataset No.	a/Å	b/Å	c/Å	β/°
**1**	30.642	14.298	6.337	94.595
**2**	30.703	14.618	6.301	98.178
**3**	31.600	14.473	6.180	96.047
**4**	33.089	13.965	6.403	94.299
**5**	31.431	14.415	6.297	96.605
**6**	31.148	14.604	6.203	94.351
**7**	31.618	14.399	6.280	98.380
**8**	31.380	14.395	6.248	93.439
**9**	31.661	14.517	6.294	96.629
**10**	31.802	14.429	6.249	95.930

* The unit cell parameters listed in the table represent the individual 3D ED datasets collected from different anhydrous sodium valproate crystals.

Table S2 Structure solution and refinement statistics of anhydrous sodium valproate.

**Method**	*c*RED
**Crystal size in diameter (μm)**	1×1×10 (needle-like)
**No. of data sets merged**	10
**Wavelength (Å)**	0.019687
**Resolution (Å)**	0.80
**Empirical formula**	$C_{24}O_6Na_3H_{45}$
**Crystal system**	Monoclinic
**Space group**	*Cc* (No. 9)
**Unit cell *a*, *b*, *c* (Å)**	31.06(6), 14.36(3), 6.24(12)
***β* (°)**	95.28(3)
**No. unique reflection ($F > 4\sigma(F)$/all)**	2038/4690
**completeness**	87.1
**$CC_{1/2}$**	95.7
**No. of atoms per asymmetric unit (non-hydrogen)**	33
**No. of Parameters**	139
**No. of Restraints**	23
**$R_1(F > 4\sigma(F))$/ R_1(all)**	0.2409/0.3256
**R_{int}**	0.2690
**GOF**	0.983
