# Supplementary material for: Unveiling the Structure of Anhydrous Sodium Valproate with 3D Electron Diffraction and a Facile Sample Preparation Workflow
Source: ACS Cent Sci. 2025 May 21;11(6):960–6. doi: 10.1021/acscentsci.5c00412 (PMC12203429; doi:10.1021/acscentsci.5c00412)
Supplement: Supplementary file 3 [file oc5c00412_si_003.pdf]

oc-2025-00412r.R1

Name: Peer Review Information for "Unveiling the Structure of Anhydrous Sodium Valproate with 3D Electron Diffraction and a Facile Sample Preparation Workflow"

First Round of Reviewer Comments

Reviewer: 1

Comments to the Author

This manuscript presents a well-executed study on the structural determination of anhydrous sodium valproate using 3D-ED coupled with a tailored glovebox-assisted plunge-freezing workflow. The work addresses a longstanding challenge in characterizing beam- and air-sensitive pharmaceutical materials and provides a generalizable methodology that could benefit a broader scientific community. The manuscript is well-written, the experimental design is rigorous, and the results are clearly presented.

I believe this work represents a significant advance in both pharmaceutical crystallography and 3D-ED methodology. I have only minor comments that should be addressed before publication.

Minor Comments and Suggestions:

1. The authors mention that seven polymorphs of sodium valproate have been reported. It would enhance the impact of the study to briefly discuss whether the current workflow could be extended to isolate or screen for other polymorphs under controlled humidity and temperature.

2. The authors report refinement using isotropic displacement parameters. Given the quality of data and cryo conditions, is there any possibility of anisotropic refinement, at least for the heavier atoms (e.g., sodium)?

3. The video showing the plunge-freezing workflow (Video S1) is highly valuable. Consider referencing it earlier in the main text (e.g., during the initial workflow description) to guide the reader.

4. Minor language corrections:

o In the Abstract: “…a feature of Na-valproate polyhedral chains.” → consider rephrasing to “…revealing the formation of Na-valproate polyhedral chains.”

o Page 4: “…the plunge-frozen gird was then placed…” → “grid” is likely intended.

Overall, this manuscript presents a technically sound and conceptually significant contribution to structural chemistry and electron diffraction methodology. With minor clarifications, this work will be suitable for publication in ACS Central Science.

Reviewer: 2

Comments to the Author

My review and Comments

[1] Page 1, Lines 31-36
If it is difficult to synthesise the crystal in sufficient size for structural study by SCXRD, then powder XRD (PXRD) or EM approach will be natural. Please give a reason why PXRD approach is excluded.

Powder sample can be put into glass-capillary and sealed inside glove-box and it will be nice to provide PXRD pattern in transmission mode.

[2] Page 3, Line 58:
Question: From the statement “Beyond its application to sodium valproate, this workflow is broadly applicable to other air-sensitive materials, including battery components, semiconductors, and biological sample.”, it is not clear how important to use “cryo-technique” for this study. If I understand correctly, EM centre, Stockholm University, has already a setup, though not “cryo-”: nanoparticles can be mount on TEM holder in a glove-box and the holder can be transferred/inserted into an EM without exposing air.

[3] Page 6, Line 50 – Page 7, Line 5:
[i] Crystal system is monoclinic (b: unique is chosen) and therefore Laue class is $2/m$. From observed conditions for reflection, possible space group is either Cc or $C2/c$. Please give the reason precisely why non-centrosymmetric Cc was chosen. This is critical for the whole discussion.

[ii] Please describe how electron multiple scattering effects on the integrated diffraction intensities of reflections are handled during the structure refinement.

[4] Page 7, Line 42:

“The unit cell axes a^*, b^* and c^* are highlighted…” should be “The unit cell parameters in reciprocal space, a^*, b^* and c^* are highlighted…”

Author's Response to Peer Review Comments:

Manuscript ID: oc-2025-00412r

Title: *Unveiling the Structure of Anhydrous Sodium Valproate with 3D Electron Diffraction and a Facile Sample Preparation Workflow*

Point-to-Point Response to the Reviewers' comments

We would like to give our sincere thanks to the Editor and Referees for the valuable comments. Please find the point-to-point responses below. The comments are given in italic. The changes and new text in the revised manuscript are highlighted in yellow.

Reviewer #1

Comment [1]

The authors mention that seven polymorphs of sodium valproate have been reported. It would enhance the impact of the study to briefly discuss whether the current workflow could be extended to isolate or screen for other polymorphs under controlled humidity and temperature.

Response:

We thank the reviewer for this insightful suggestion. Indeed, the workflow combining plunge-freezing and 3D electron diffraction could potentially be extended for the isolation or screening of other polymorphs of sodium valproate, particularly those that are metastable or sensitive at ambient conditions. We have now briefly discussed this possibility in the revised manuscript.

Page 11, Last paragraph was changed from:

"This workflow not only facilitated the successful structural determination of anhydrous sodium valproate, but also offers the potential for the structure determination of other polymorphs of sodium valproate in the future."

to

"This workflow not only facilitated the successful structural determination of anhydrous sodium valproate, but also offers the potential for the discovery and structure determination of other polymorphs. When combined with controlled crystallization conditions, such as variations in humidity and temperature, this approach could enable the isolation and structural characterization of additional sodium valproate polymorphs, including metastable forms that are otherwise challenging to capture."

Comment [2]

The authors report refinement using isotropic displacement parameters. Given the quality of data and cryo conditions, is there any possibility of anisotropic refinement, at least for the heavier atoms (e.g., sodium)?

Response:

We appreciate the reviewer's suggestion. We attempted anisotropic refinement for the non-hydrogen atoms or heavy atoms only (Na atoms). However, due to the relatively high mosaicity of the crystals and the limitations introduced by dynamical scattering in 3D ED data, the anisotropic refinement did not yield chemically reasonable anisotropic displacement parameters. We therefore report isotropic displacement parameters, which provided a more stable and chemically reasonable model. This point has now been clarified in the revised manuscript.

Page 12, first paragraph was changed from:

"The initial model of sodium valproate was solved from the merged dataset by dual-space methods using SHELXT[28], which was further refined isotropic atomic displacement parameters using SHELXL[30] and ShelXle[29]."

to

"The initial model of sodium valproate was solved from the merged dataset by dual-space methods using SHELXT[28]. However, due to the relatively high mosaicity of the crystals and the limitations introduced by dynamical scattering in 3D ED data, the refinement did not yield chemically meaningful anisotropic displacement parameters. Therefore, the structure was refined using SHELXL[30] and ShelXle[29] with isotropic atomic displacement parameters."

Comment [3]

The video showing the plunge-freezing workflow (Video S1) is highly valuable. Consider referencing it earlier in the main text (e.g., during the initial workflow description) to guide the reader.

Response:

Thank you for the helpful comment. We have now added a reference to Video S1 earlier in the Introduction section to better guide the reader.

Page 3, Last paragraph was changed from:

"In this study, a nitrogen-regulated glovebox equipped with a cooling chamber was designed to enable plunge freezing[27] in a controlled atmosphere (**Figure 2, Figures S1 and S2**)."

to

"In this study, a nitrogen-regulated glovebox **(Figure 2)** equipped with a cooling chamber was designed to enable plunge freezing[27] in a controlled atmosphere **(Video S1)**."

Comment [4]

- *In the Abstract: "…a feature of Na-valproate polyhedral chains." → consider rephrasing to "…revealing the formation of Na-valproate polyhedral chains."*
- *Page 4: "…the plunge-frozen gird was then placed…" → "grid" is likely intended.*

Response:

Thank you for pointing out these language issues. We have corrected "gird" to "grid" on **Page 4 (page 5 in the new version)**, and rephrased the sentence in the **Abstract** to "…revealing the formation of Na-valproate polyhedral chains." in the revised manuscript.

Reviewer #2

Comment [1] (Page 1, Lines 31–36):

If it is difficult to synthesise the crystal in sufficient size for structural study by SCXRD, then powder XRD (PXRD) or EM approach will be natural. Please give a reason why PXRD approach is excluded. Powder sample can be put into glass-capillary and sealed inside glove-box and it will be nice to provide PXRD pattern in transmission mode.

Response:

We thank the reviewer for this valuable suggestion. The PXRD pattern was measured using an in-house diffractometer and shows significant peak broadening and overlapping, which complicate reliable intensity extraction required for ab initio structure determination. These issues arise even though the crystals are several micrometres in length, suggesting high mosaicity. We have now included a brief explanation of this in the revised manuscript.
We have added the following discussions **on Page 2, second paragraph**:

"Furthermore, the PXRD pattern collected under dry conditions (Figure S1, Supporting information with experimental details provided in the Methods session) shows peak broadening and overlapping, making *ab initio* structure solution of anhydrous sodium valproate by PXRD challenging."

In addition, the PXRD pattern of anhydrous sodium valproate is included in the Supporting Information (Figure S1) for reference. Due to the addition of a new supporting figure, the PXRD pattern originally presented as Figure S1 and S2 is now updated to Figure S2 and S3 in the revised Supporting Information.

The experimental details are added in the **Methods section**:

"**Power X-ray diffraction**
Powder X-ray diffraction (PXRD) data were collected on a PANalytical X'Pert PRO diffractometer equipped with a Cu Kα radiation source (λ = 1.5406 Å) operating at 45 kV and 40 mA. The measurements were performed in reflection mode using a PW3064/60 spinner

stage. Data were recorded over a 2θ range of 5.00–35.00° with a step size of 0.0167° and a continuous scan mode. A fixed divergence slit of 0.10 mm was used."

Reviewer Comment [2] (Page 3, Line 58):

From the statement "Beyond its application to sodium valproate, this workflow is broadly applicable to other air-sensitive materials..." it is not clear how important to use "cryo-technique" for this study. If I understand correctly, EM centre, Stockholm University, has already a setup, though not "cryo-": nanoparticles can be mount on TEM holder in a glove-box and the holder can be transferred/inserted into an EM without exposing air.

Response:

We appreciate the reviewer's insightful observation. It is correct that at the Stockholm University EM Centre, a room-temperature vacuum transfer TEM holder is available and can be operated in a glove box. However, in our study, cryogenic condition was essential not only to prevent air exposure but also to mitigate electron beam damage during data collection. A clarification was added in **Page 4, first paragraph**:

"As many of these materials are also highly susceptible to electron beam damage, the use of cryogenic protection is essential to minimize beam-induced degradation during 3D ED data collection."

Reviewer Comment [3i] (Page 6, Line 50 – Page 7, Line 5):

Crystal system is monoclinic (b: unique is chosen) and therefore Laue class is 2/m. From observed conditions for reflection, possible space group is either Cc or C2/c. Please give the reason precisely why non-centrosymmetric Cc was chosen. This is critical for the whole discussion.

Response:

Thank you for pointing this out. Based on the systematic absence conditions, both *Cc* and *C*2/*c* were initially considered. Structure solution attempts in *C*2/*c* failed to yield a chemically reasonable model, whereas the solution in *Cc* resulted in a consistent atomic structure with acceptable geometry and refinement residuals. Additionally, refinement in *C*2/*c* introduced disorder across a symmetry element, which was resolved when the space group was lowered to *Cc*. As shown below, the structure model refined in *C*2/*c* contains clashes/disorder due to the additional inversion centre. The atoms in this region exhibit unreasonable bond lengths and bond angles, indicating that *C*2/*c* is not the correct space group for this structure. (Figure R1)

In addition, there is no centre of symmetry in our structural model. The choice of the non-centrosymmetric *Cc* space group is thus supported by both chemical reasoning and refinement statistics. We have now clarified this point in the revised manuscript (**Page 7, first paragraph**):

"Structure solution in the *C*2/*c* space group failed to yield a chemically reasonable model and resulted in disorder and steric clashes. In contrast, solution and refinement in the *Cc* space

group produced a chemically reasonable structural model. Accordingly, the initial structure was solved in *Cc* using the integrated intensities...”

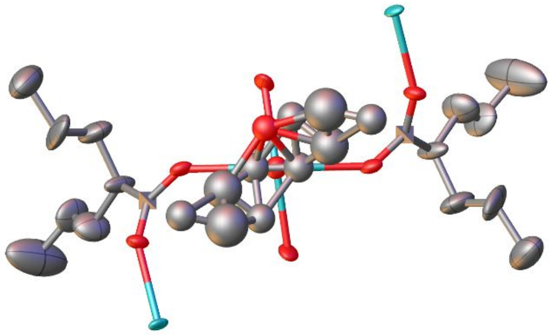

Figure R1. The structure model refined in the space group *C*2/*c*, viewed along the [010] direction.

Reviewer Comment [3ii]:

Please describe how electron multiple scattering effects on the integrated diffraction intensities of reflections are handled during the structure refinement.

Response:

During 3D ED data collection, we rotate the crystal along an arbitrary axis, which helps reduce the likelihood of multiple scattering events. In the data processing stage, we actively exclude frames acquired along major zone axes, where the probability of dynamical scattering is highest.

Sodium valproate crystals studied in this work exhibited relatively high mosaicity, as evidenced by broad peaks in the PXRD pattern. Although higher mosaicity increases the size of the reflections, it also makes it less coherent, reducing dynamical scattering effects.

Moreover, we merged 10 datasets during data processing to improve the completeness and data redundancy, while reducing random error. Individual datasets were collected on crystals over different orientations, so that the dynamical scattering conditions also differs among datasets. It has been shown before that with higher data redundancy, the integrated intensities also became more accurate due to averaging.

Finally, the main objective of this study was to determine a chemically reasonable structure of anhydrous sodium valproate. Given the sample characteristics and experimental limitations, refinement against kinematical intensities was both sufficient and appropriate.

These considerations and related details are described in the revised manuscript.

Page 12, first paragraph was added a description about data merging:

“It is worth noting that diffraction patterns collected along major zone axes were excluded during data processing to eliminate reflections severely affected by dynamical scattering. To

improve data redundancy, 3D ED datasets were merged from multiple crystals. By combining data from crystals of varying thicknesses and orientations, the accuracy of the measured reflection intensities was further enhanced[34]. "

Reviewer Comment [4] (Page 7, Line 42):
*"The unit cell axes a**, *b** and *c** are highlighted…" should be "The unit cell parameters in reciprocal space, *a**, *b** and *c** are highlighted…"

Response:
We thank the reviewer for catching this imprecision. The sentence has been corrected: *"The unit cell parameters in reciprocal space, a*, b** and *c** are highlighted..." (**Page 7, Figure 3 Title**).

Response to Formatting Requirements

We thank the editor for the helpful formatting suggestions. The manuscript and supporting documents have been revised accordingly as detailed below.

1. Supporting Information (SI) Description in the Main Manuscript

The paragraph describing the Supporting Information in the main manuscript has been revised to include a clear and complete list of all items included in the SI-for-Publication file. Each item is now individually described in a dedicated paragraph placed after the Acknowledgment section, following the recommended format.

Supporting information
Additional experimental details, including:
Figure S1 The PXRD pattern of anhydrous sodium valproate.
Figure S2 Schematic design of the nitrogen-regulated glovebox equipped with a cooling stage.
Figure S3 An photograph of the nitrogen-regulated glove box with cooling stage.
Table S1 Summary of the unit cell parameters of anhydrous sodium valproate crystals determined by 3D ED.
Table S2 Structure solution and refinement statistics of anhydrous sodium valproate.
Crystallography file CCDC 2356987 contains the crystallographic data for this paper. These data can be obtained free of charge via www.ccdc.cam.ac.uk/data_request/cif, or by emailing data_request@ccdc.cam.ac.uk, or by contacting The Cambridge Crystallographic Data Centre, 12 Union Road, Cambridge CB2 1EZ, UK; fax: +44 1223 336033.
Video S1 Workflow for cryo-sample preparation using plunge freezing in a glovebox.

2. Supporting Information Page Numbering

All pages in the SI-for-Publication file have been numbered in the format: S1, S2, S3, etc., as requested.

3. Synopsis

A brief synopsis (A cryo-transfer workflow enables 3D ED structure determination of hygroscopic sodium valproate, offering a strategy for studying materials sensitive to both electron beam and ambient air.) has been added to the main manuscript file, placed directly after the TOC graphic and labelled as Synopsis. The synopsis highlights the significance of the work and is consistent with the TOC graphic content.

4. TOC Graphic

A Table of Contents (TOC) graphic has been included on the last page of the manuscript file. The graphic is original, representative of the overall study, and follows ACS guidelines. All text in the TOC graphic is legible, and no caption has been included, in accordance with the journal's requirements.

We hope these revisions meet the formatting requirements. Please let us know if any additional adjustments are needed.

Further Revisions made:

As part of our thorough revision process, we revisited the interpretation and discussion sections and identified a few statements that were more speculative than warranted by the data. Although the reviewers did not raise this issue, we have revised these statements to enhance the scientific rigor of the manuscript.

Page3, Figure 1 Title was changed from "The details of the hygroscopicity measurements are provided in the Supporting Information." to "The details of the hygroscopicity measurements are provided in the Methods section."

Page 8, last paragraph was changed from:

"…water molecules can easily diffuse into the inter-alkyl spaces and penetrate the hydrophobic barriers when sodium valproate is exposed to air. Subsequently, owing to the hydrophilic nature of sodium ion, the water molecule can readily coordinate with the sodium clusters at the centre."

to

"…water molecules may diffuse into the inter-alkyl spaces when sodium valproate is exposed to air. Meanwhile, the hydrophilic sodium ion provides potential binding sites for water molecules."

Page 9, last paragraph was changed from:

"As shown in **Figure 6a**, the presence of a coordinated water molecule perturbs the coordination environment of sodium ion, resulting in the monohydrate sodium valproate packing with a low-symmetry space group *P*-1. Consequently, its inner sodium–oxygen cluster exhibits a looser arrangement (**Figure 6b**). During dissolution, the hydrophobic layers of monohydrate form are likely to be disrupted by the coordinated water molecule, potentially leading to a faster dissolution rate. In contrast, anhydrous sodium valproate adopts a more ordered crystalline structure with a monoclinic space group *Cc*. As shown in **Figure 6c**, the sodium–oxygen clusters in the anhydrous form exhibit a more compact and orderly atomic

arrangement, resulting in a higher crystallographic density of 1.195 g/cm^3, compared to 1.099 g/cm^3 for the monohydrate form. The denser and more hydrophilic packing of anhydrous sodium valproate indicates a dynamically less favourable structure in aqueous solution, which may contribute to a slower dissolution rate compared to the monohydrate form."

to

"As shown in **Figure 6a**, the monohydrate form crystallizes in the triclinic space group *P*-1, with a water molecule coordinating to the sodium ion. Similar to the anhydrous form, the monohydrate form is stabilized by van der Waals interactions between the alkyl chains of valproate molecules. The sodium–oxygen coordination clusters of monohydrate form and anhydrous form are shown in **Figure 6b** and **6c**, respectively. The anhydrous form exhibits a more compact and regular atomic arrangement compared to the monohydrate form. The calculated density of the anhydrous form is 1.195 g/cm3, whereas the monohydrate form has a lower density of 1.099 g/cm3."

The affiliation of the last author, Hongyi Xu, has also been updated. He is now a senior lecturer at the Australian National University. This information has been added to the affiliation section.

"d Research School of Chemistry, Australian National University, Acton, ACT 2601, Australia."
